# Sex Differences in the Clinical Recognition of Significant Aortic Stenosis

**DOI:** 10.1016/j.jacadv.2025.102370

**Published:** 2025-11-25

**Authors:** Daniela Crousillat, Dayana Shariff, Rong Duan, Varsha Tanguturi, Sammy Elmariah

**Affiliations:** a Division of Cardiovascular Science, Department of Medicine and Obstetrics and Gynecology, University of South Florida Morsani College of Medicine, Tampa General Heart and Vascular Institute, Tampa, Florida, USA; b University of California, San Francisco School of Medicine, San Francisco, California, USA; c Division of Cardiology, University of California, San Francisco School of Medicine, San Francisco, California, USA; d Division of Cardiology, Massachusetts General Hospital, Boston, Massachusetts, USA

**Keywords:** aortic stenosis, diagnosis, disparity, equity, female, sex

## Abstract

**Background:**

Women with significant aortic stenosis (AS) are under-represented among patients undergoing aortic valve replacement in the United States as compared with men despite similar disease prevalence.

**Objectives:**

This study evaluated the impact of sex on the clinical recognition of significant AS following diagnostic transthoracic echocardiogram (TTE).

**Methods:**

Using data from a large administrative multicenter database, we assessed rates of clinical diagnosis of AS defined by assignment of an Internal Classification Diagnosis (ICD) code for AS following a diagnostic TTE. Fine-Gray models were used to evaluate the 1-year cumulative rate of AS diagnosis by sex, AS severity, and AS phenotype accounting for competing risk of death.

**Results:**

Among 2,036 included patients (48.9% female, 51.1% male), the 1-year rate of clinical recognition following a diagnostic TTE with at least moderate AS was low (52.4%), and women were less likely as compared with men to receive a clinical diagnosis of AS (55.7% male vs 48.9% female; *P* = 0.003). Specifically, among patients with severe AS, women were less likely to receive an ICD diagnosis as compared with males (64.1% vs 74.3%), driven primarily by lower rates of clinical recognition of severe low gradient AS (66.9% male vs 54.3% female; *P* = 0.004).

**Conclusions:**

Rates of clinical recognition of AS following a diagnostic TTE are low, and vary significantly by sex, AS disease severity, and severe AS phenotype. Further studies are needed to maximize the clinical recognition of TTE-confirmed AS and to mitigate sex disparities in diagnosis and treatment.

Aortic stenosis (AS) is similarly prevalent in both men and women,^1^ yet significant sex differences exist in the pathophysiology, management, and outcomes of AS. Women with severe AS are generally older, present with higher symptomatic burden, and are more likely to present with discordant low gradient severe AS. Women have a higher mortality following AS diagnosis as compared with men, in part due to delayed diagnosis and lower rates of aortic valve replacement (AVR) despite similar disease severity.^2^^,^^3^ Although surgical AVR is traditionally associated with a higher perioperative mortality in women as compared with men, transcatheter aortic valve implantation has demonstrated a female-specific survival advantage over surgical AVR,^4^ elevating the importance of timely diagnosis and treatment of significant AS.^5^, ^6^, ^7^, ^8^ Women are less likely to meet the guideline criteria for valvular intervention^9^ for severe AS due to anatomical and pathophysiological differences including higher left ventricular (LV) ejection fraction (LVEF) and prevalence of paradoxical low-flow, low-gradient AS which fail to meet the standard guidelines for valvular intervention. The pathobiology of aortic valve disease in women is driven primarily by fibrotic remodeling of the valve as compared to valvular calcification which predominates among men, potentially affecting hemodynamic assessment.^10^ No significant sex differential in the hemodynamic progression of AS has been previously demonstrated,^11^^,^^12^ but emerging data support female sex as an independent determinant of faster AS progression associated with higher AS-related event rates.^13^ Despite these differences in pathophysiology, hemodynamic progression, and discordant phenotypes, current clinical guidelines do not include sex-specific recommendations for the diagnosis or treatment of AS.^14^

The historical absence of standardized echocardiography reporting for AS and quality metrics evaluating the entire continuum of care from echocardiographic diagnosis to treatment of significant AS has limited our understanding of contributors to sex disparities in AS care. Limited data exist on sex differences in the recognition of clinically significant AS following diagnostic transthoracic echocardiogram (TTE), a critical first step in the initiation of AS care. Routine echocardiography reporting practice at our institution includes review and confirmation of echocardiography findings by a cardiologist, after which results are automatically sent to the ordering provider and available for review in the electronic health record (EHR). We sought to assess how often a TTE-confirmed diagnosis of moderate or greater AS led to provider-led recognition of AS by issuance of an AS-related diagnosis code within 12 months following the TTE. Using the Mass General Brigham Research Patient Data Registry (RPDR), we previously reported low rates of clinical recognition of AS based on Internal Classification Diagnosis (ICD) coding following diagnostic TTE among patients with any severity of AS. Female sex was independently associated with a lower likelihood of receiving an AS diagnosis within the year following TTE among patients with any severity of AS.^15^ We sought to further characterize these sex-based differences among individuals with significant (greater than moderate) AS to better assess the contributors to delayed diagnosis.

## Methods

### Study population

**Figure 1:**
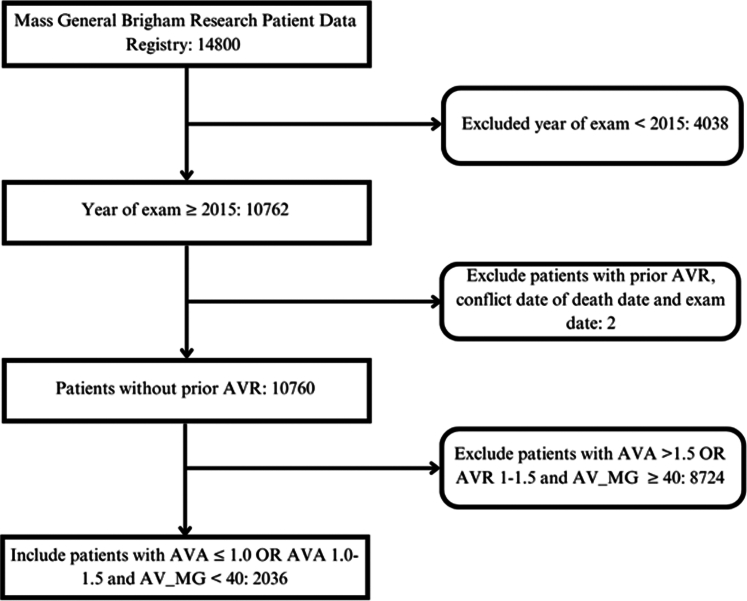
**Study Protocol** We identified 2036 TTEs between 2015 and 2020 with moderate or greater severity of AS based on AVG or MG without an existing diagnosis of AS to assess the 1-year rates of AS ICD diagnosis following index TTE. AVA = aortic valve area; AV_MG = transvalvular mean pressure gradient; AVR = aortic valve replacement; TTE = transthoracic echocardiogram.

We obtained an abstract of TTE reports that took place between 2015 and May 2020 from the RPDR, an administrative multicenter database for our health care system encompassing 2 large academic centers, Massachusetts General Hospital and Brigham and Women’s Hospital. We identified patients with AS for whom a transvalvular mean pressure gradient (MG) and aortic valve area (AVA) was reported. We defined AS severity by AVA and MG based on the following echocardiographic diagnostic criteria established by the American College of Cardiology/American Heart Association valvular heart disease guidelines^14^ moderate AS (MG <40 mm Hg and AVA >1 cm^2^ and <1.5 cm^2^), severe high gradient AS (MG >40 mm Hg and AVA <1 cm^2^), and severe low gradient AS (AVA <1 cm^2^ and MG <40 mm Hg).^16^ Records were chronologically sorted to identify an index date for each patient (ie, the first-known date where a MG and AVA was reported). Histories of medical encounters and diagnoses were analyzed to identify the earliest date, if any, in which a diagnosis code for AS (ICD-9 code: 424.1; ICD-10 code: I35.0) was issued to each patient. Patients with an ICD diagnosis for AS before the date of their index TTE were excluded from this study as were patients with prior surgical aortic valve replacement/transcatheter aortic valve implantation. Patients for whom “other” or “unknown” was indicated as self-reported sex and patients under the age of 18 were excluded. In addition, patients with year of exam before 2015 and patients who had conflicts between date of death and exam dates were excluded (Figure 1). This retrospective cohort study was approved by the Mass General Brigham Institutional Review Board before data collection and analysis.

### Study variables

The primary outcome was defined by the documentation of an AS ICD-9 or -10 diagnosis code in the EHR following index TTE demonstrating at least moderate AS. Independent variables used in our analysis included sex, race/ethnicity, age, primary language, residential setting, type of insurance carrier, AS severity, combined comorbidity index (CCI) score, ordering provider specialty, and TTE setting. Categories for sex (defined as sex assigned at birth) were self-reported by patients as male or female, gender was not available, and all reported analysis are based on self-reported sex. Age, race/ethnicity, primary language, and primary insurance were extracted from the EHR. Patient comorbidities at the time of their index TTE were assessed using the CCI score.^17^ We matched patient zip codes contained in their EHRs to urban cluster area files from the U.S. Census Department to determine whether each patient was living in a primarily urban or nonurban setting. We identified urban clusters linked to each zip code and used the total zip code population percentage column in the Census area files to classify each patient as primarily urban (>50% of the total population in the patient’s zip code lived within urban limits) or rural (<50% of the total population in the patient’s zip code lived within urban limits). For ordering providers associated with Mass General Brigham institutions, medical specialty was obtained from provider registration information provided by RPDR. For external providers, specialty was obtained through an electronic query of the Centers for Medicare and Medicaid Services National Provider Identification registry. The setting where the TTE was performed (ie, inpatient vs outpatient) was determined using the associated medical encounter type in EHR.

### Statistical analysis

Baseline characteristics and TTE data were summarized using scale-appropriate measures for categorical variables (eg, counts, percentages) and continuous variables (eg, mean ± SD or median with 25th-75th percentiles [Q1-Q3]). Differences in categorical variables were ascertained using the chi-square test, whereas the Welch t-test was used for comparing continuous variables. The Gray test cumulative incidence function (CIF) curves were used to assess the 1-year cumulative rate of AS diagnosis stratified by severity, gradient, and sex including death as a competing risk^18^^,^^19^ We constructed a Fine-Gray subdistribution hazard model to determine multivariable associations between sex and cumulative probability of 1-year AS diagnosis. Fine-Gray models were adjusted for age, race, sex, primary language, residential setting, type of insurance carrier, AS severity, CCI score, ordering provider specialty, and TTE setting. CIF curves and subdistribution HRs (sHRs) are reported with corresponding 95% CIs. Probability thresholds for statistical significance were set at 0.05 using 2-sided tests; all analyses were conducted using R (4.3.3) statistical programming software.

## Results

### Patient characteristics

Among 2,036 included patients, 48.9% female, 51.1% male, 63.8% of patients had moderate AS, and 36.2% severe AS. The proportion of patients within each AS severity category, including high and low gradient severe AS phenotypes, was similar by sex. Among the entire study population, 45.0% of individuals were aged >80 (*P* < 0.001) years, of which women represented a larger proportion (49.3% female vs 41.0% male). Both groups had equal burden of comorbidities, mostly resided in an urban setting, and were most frequently insured by Medicare. Both groups were equally as likely to obtain TTEs in the outpatient setting (82.7% vs 17.3% inpatient) of which the majority (75.4%) were ordered by noncardiologist with no significant difference in the ordering provider by sex. There were no sex differences in 1-year mortality following diagnostic TTE (male 6.5% vs female 6.7%). Differences in the prevalence of demographic characteristics and clinical risk factors by sex are shown in Table 1.

**Table 1:** Baseline Characteristics

	Overall (N = 2,036)	Female (n = 996)	Male (n = 1,040)	*P* Value^a^
Age (years) at exam, n (%)				<0.001
<65	312 (15.3%)	140 (14.1%)	172 (16.5%)	
65-80	807 (39.6%)	365 (36.6%)	442 (42.5%)	
>80	917 (45.0%)	491 (49.3%)	426 (41.0%)	
Race, n (%)				0.56
Non-Hispanic White	1,900 (93.3%)	923 (92.7%)	977 (93.9%)	
Non-Hispanic Black	70 (3.4%)	36 (3.6%)	34 (3.3%)	
Hispanic	35 (1.7%)	21 (2.1%)	14 (1.3%)	
Non-Hispanic Asian	31 (1.5%)	16 (1.6%)	15 (1.4%)	
English, n (%)				0.98
English	1,884 (92.5%)	921 (92.5%)	963 (92.6%)	
Non-English	152 (7.5%)	75 (7.5%)	77 (7.4%)	
Urban, n (%)				0.35
Urban	1,908 (93.7%)	939 (94.3%)	969 (93.2%)	
Not-urban	128 (6.3%)	57 (5.7%)	71 (6.8%)	
Cardiologist, n (%)				0.18
Cardiologist	500 (24.6%)	231 (23.2%)	269 (25.9%)	
Non-cardiologist	1,536 (75.4%)	765 (76.8%)	771 (74.1%)	
TTE setting, n (%)				0.94
Outpatient	1,684 (82.7%)	825 (82.8%)	859 (82.6%)	
Inpatient	352 (17.3%)	171 (17.2%)	181 (17.4%)	
Insurance, n (%)				0.086
Medicaid/MassHealth	60 (2.9%)	26 (2.6%)	34 (3.3%)	
Medicare	1,334 (65.5%)	676 (67.9%)	658 (63.3%)	
Other	642 (31.5%)	294 (29.5%)	348 (33.5%)	
CCI score n (%)				0.81
<5	1,123 (55.2%)	542 (54.4%)	581 (55.9%)	
5-9	576 (28.3%)	286 (28.7%)	290 (27.9%)	
≥10	337 (16.6%)	168 (16.9%)	169 (16.3%)	
Hypertension, n (%)	1,076 (52.8%)	532 (53.4%)	544 (52.3%)	0.65
Complicated diabetes, n (%)	385 (18.9%)	171 (17.2%)	214 (20.6%)	0.057
Congestive heart failure, n (%)	761 (37.4%)	366 (36.7%)	395 (38.0%)	0.60
Renal failure, n (%)	491 (24.1%)	206 (20.7%)	285 (27.4%)	<0.001
AS severity based on AVA, n (%)				0.75
Moderate	1,298 (63.8%)	639 (64.2%)	659 (63.4%)	
Severe	738 (36.2%)	357 (35.8%)	381 (36.6%)	
Aortic valve area (cm^2^)				0.33
Mean (SD)	1.1 (0.3)	1.1 (0.3)	1.1 (0.3)	
Median (Q1, Q3)	1.2 (0.9, 1.3)	1.1 (0.9, 1.3)	1.2 (0.9, 1.3)	
AV mean pressure gradient (mm Hg)				0.002
Mean (SD)	22.2 (13.4)	21.3 (13.8)	23.1 (12.9)	
Median (Q1, Q3)	19.0 (13.0, 28.0)	18.0 (12.0, 27.0)	20.3 (14.0, 29.0)	
AS severity based on gradient, n (%)				0.93
Moderate	1,298 (63.8%)	639 (64.2%)	659 (63.4%)	
Severe low-gradient	528 (25.9%)	256 (25.7%)	272 (26.2%)	
Severe high-gradient	210 (10.3%)	101 (10.1%)	109 (10.5%)	

AS = aortic stenosis; AVA = aortic valve area; AV = aortic valve; CCI = combined comorbidity index; TTE = transthoracic echocardiogram.

a Pearson chi-square test; Welch t-test.

### Sex differences in cumulative 1-year rates of AS diagnosis stratified by disease severity and AS phenotype

**Figure 2:**
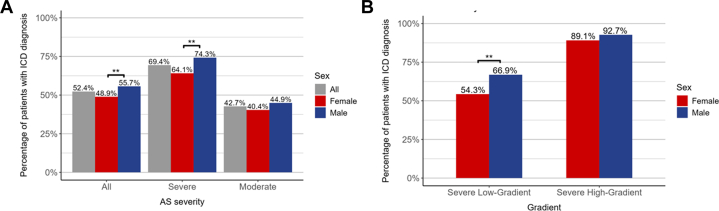
AS Diagnosis Rates at 12 Months A Unadjusted rates of diagnosis for all AS, severe AS, and moderate AS stratified by sex (A) and gradient (B). Severe high-gradient AS defined by AVA <1 cm^2^ and MG >40 mm Hg. Severe low-gradient AS defined (typo) as AVA <1 cm^2^ and MG <40 mm Hg. Severe low-gradient AS defines as: AVA <1 cm^2^ and MG <40 mm Hg. ∗∗*P* < 0.01. AS = aortic stenosis; ICD = internal classification diagnosis.

**Figure 3:**
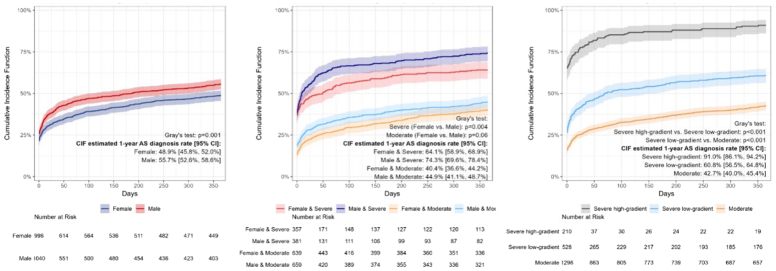
Cumulative Incidence Function of AS Diagnosis at 12 Months CIF curves for AS diagnosis by sex (A), severity (B), and gradient (C). CIF = cumulative incidence function; other abbreviations as in Figure 2.

Among patients with at least moderate AS on TTE, 52.4% received an ICD diagnosis of AS within 1 year of index TTE. There were significant sex differences in the 1-year rates of clinical recognition of AS following index TTE. Among the entire cohort, men were more likely to receive an ICD diagnosis for AS compared to women (55.7% male vs 48.9% female; *P* = 0.003) (Figure 2A). Stratified by disease severity, 69.4% of individuals with severe AS received a diagnosis compared with 42.7% of individuals with moderate AS. Stratified by sex, the 1-year unadjusted rates of AS ICD diagnosis varied significantly for severe AS (74.3% male vs 64.1% female; *P* = 0.004). There were no sex differences in the rates of receiving a diagnosis for moderate AS (44.9% male vs 40.4% female; *P* = 0.11) (Figure 2A). Stratified by sex and severe AS phenotype (low gradient vs high gradient), there were no sex differenecs in AS diagnosis for severe high gradient AS (92.7% male vs 89.1% female, *P* = 0.51). Rates of AS ICD diagnosis varied significantly by sex among individuals with severe low gradient AS (66.9% male vs 54.3% female; *P* = 0.004) (Figure 2B). The CIF curves for diagnosis by sex, AS severity, and gradient are shown in Figure 3.

### Predictors of cumulative 1-year rates of diagnosis

**Figure 4:**
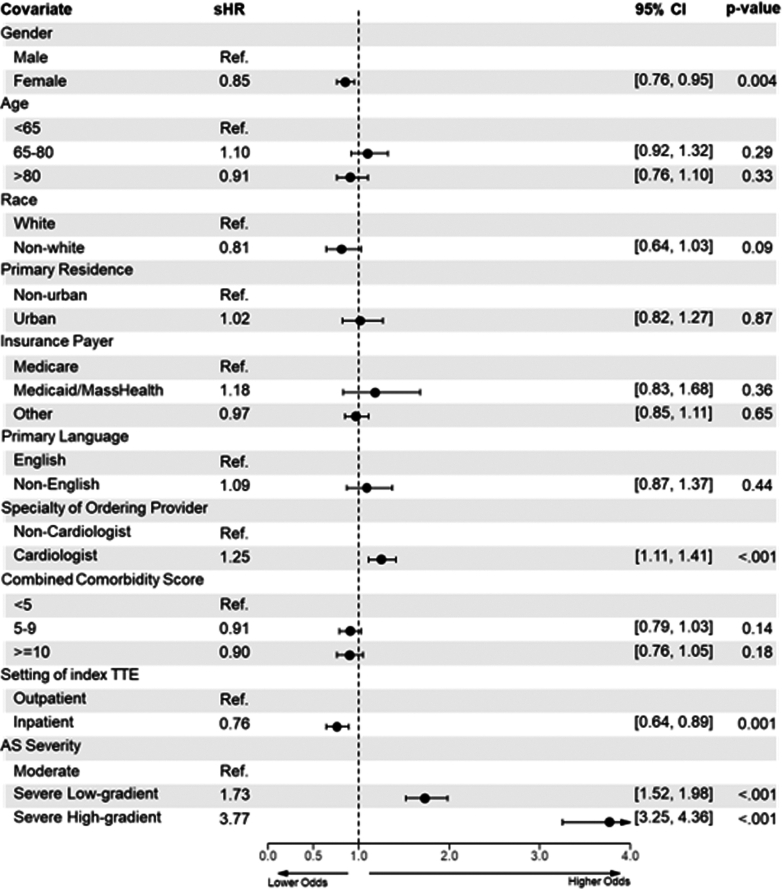
**Multivariable Regression Model Between Sex and 1-Year Aortic Stenosis Diagnosis** sHR = subdistribution HR; TTE = transthoracic echocardiogram; other abbreviations as in Figure 2.

In a multivariable Fine-Gray model analysis, sex was a significant predictor of receiving an ICD diagnosis of AS. Compared to male sex, female sex was associated with a lower likelihood of a diagnosis of AS following TTE (sHR: 0.85; 95% CI: 0.76-0.95; *P* = 0.004). Compared to moderate AS, severe AS independent of gradient was associated with a greater likelihood of receiving a diagnosis, a finding that was most significant for a high gradient severe AS (high gradient: sHR: 3.77; 95% CI: 3.25-4.36; *P* < 0.001) (Figure 4). A cardiologist as the ordering provider for TTE was found to have a positive association with receiving an AS diagnosis (sHR: 1.25; 95% CI: 1.11-1.41; *P* < 0.001) while obtaining a diagnostic TTE in the inpatient setting was associated with lower probability of receiving a diagnosis (sHR: 0.76; 95% CI: 0.64-0.89; *P* = 0.001).

**Central Illustration:**
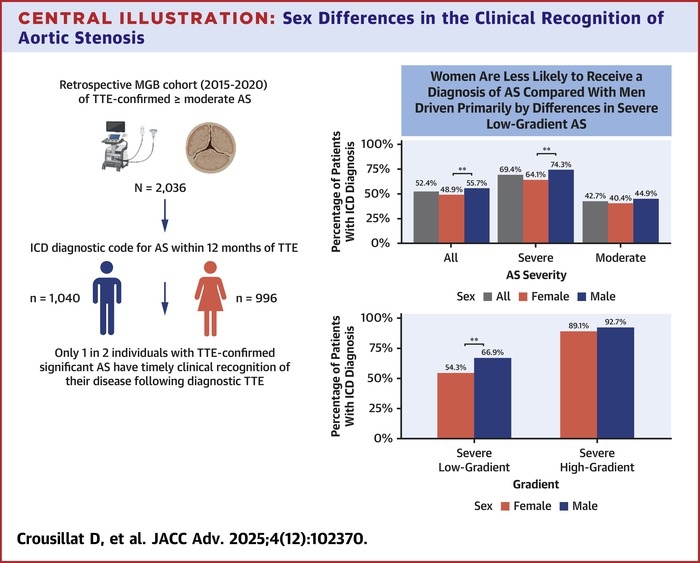
**Sex Differences in the Clinical Recognition of Aortic Stenosis** Rates of clinical recognition of AS following a diagnostic TTE are low, and vary significantly by sex, AS disease severity, and severe AS phenotype. Further studies are needed to maximize the clinical recognition of TTE-confirmed AS and to mitigate sex disparities in diagnosis and treatment. AS = aortic stenosis; ICD = internal classification diagnosis; MGB = Mass General Brigham; TTE = transthoracic echocardiogram.

## Discussion

Among individuals with moderate or greater AS diagnosed by TTE, we demonstrated differential rates of AS recognition by sex, disease severity, and AS phenotype. The recognition of significant AS confirmed on echocardiography was low among all individuals independent of sex or AS severity. Women, as compared with men, were less likely to receive a diagnosis code for severe AS, with lowest rates among women with severe low gradient AS. There were no sex differences in the recognition of moderate or severe high gradient AS. Our findings highlight important sex disparities in the timely identification and clinical recognition of AS with important implications for improving known sex disparities in the treatment of AS.

Approximately half of all individuals with a TTE that was diagnostic for moderate or severe AS did not receive a clinical ICD diagnosis code for AS within 12 months. There was an increase in rates of diagnosis with increasing AS disease severity; however, a substantial diagnosis disparity gap persisted. Our study results add to the understanding of well-documented sex disparities in the undertreatment of severe AS and are importantly the first to report clinical under recognition of significant AS by sex based on diagnostic coding.^20^, ^21^, ^22^ Women generally present at older age with more advanced stages of disease and are least likely to receive an AS diagnosis or undergo AVR.^23^ This is in context of low rates of AVR among all symptomatic severe AS patients with Class I indications for AVR, of which two-thirds undergo intervention compared to one-third with low gradient AS phenotypes which are more likely to remain under-recognized and untreated. The clinical importance of timely diagnosis and treatment for all patients with severe AS, regardless of sex, cannot be sufficiently stated. The recent Target AS pilot initiative supported by the American Heart Association is leveraging institutional data to establish foundational efforts to improve AS care outcomes through systematic evaluation of clinical processes following initial echocardiographic detection of AS. Quality care metrics including standardized reporting of qualitative and quantitative AS severity metrics by TTE, time to repeat TTE when surveillance is appropriate, and time to treatment for patients with Class I indications for AVR will track institutional compliance with guideline based care to help address undertreatment as well as delays in AS care.^24^

Low gradient severe AS phenotypes and discordant AS findings on echocardiography pose an additional diagnostic challenge to the appropriate recognition and diagnosis of clinically significant AS. The presence of coexistent heart failure independent of LVEF is the most common cause of lower stroke volume, transaortic gradients, and increased risk for underestimation of AS severity. Women are more likely to have low gradient AS phenotypes compared to men secondary to sex differences in adaptive response to pressure overload characterized by concentric LV hypertrophy, smaller LV cavity size, and preserved LVEF.^25^, ^26^, ^27^ In addition, women have smaller aortic valve annulus corresponding to smaller body surface area and higher stroke volume index;^28^ however, current guidelines lack sex-specific definitions for AS^25^ or sex-specific treatment thresholds for valvular intervention. The guideline definition of AS relies on peak velocity and mean gradient thresholds, thus biasing toward the diagnosis of high gradient AS, a phenotype less common among women.^16^ In our study cohort, low gradient severe AS was present equally based on sex; however, only half of women with severe low gradient AS obtained an ICD AS diagnosis by 12 months, a finding that was significantly lower compared to men with the same AS phenotype. The under appreciation of AS severity on TTE is insufficient to explain low diagnosis rates given the persistent sex disparity even when accounting for the same AS phenotype. Women with discordant low gradient severe AS have worse mortality and are less likely to be referred to AVR compared with men with similar presentation.^29^ Although men and women had similar rates of congestive heart failure, men with low gradient severe AS are more likely to have impaired LV dysfunction (LVEF <50%) which is an additional indication for AVR in context of severe AS and may explain at least in part the lower likelihood of women to be treated for AS compared with men. We call for the continued study of sex variability in the diagnostic thresholds used by echocardiography to define AS among women as well as sex-specific considerations for valvular intervention that reflect differences in AS pathophysiology, phenotype, and outcomes.

The appropriate recognition and diagnosis of significant AS by echocardiography represents a primordial and essential step in cascade of appropriate downstream treatment strategies. No current echocardiography reporting standards exist regarding the inclusion of a summative assessment of AS severity or recommendations regarding a follow-up plan among patients identified to have severe AS. Our study findings reflect gaps in access to subspecialty care (only 25% of TTEs were ordered by cardiologists) and significant delays in the recognition of significant AS. We hypothesize these gaps may lead to delays in appropriate referral of significant AS as the majority of TTEs are ordered by noncardiology providers who may be less familiar with diagnostic echocardiographic parameters and treatment guidelines, particularly for cases with discordant findings which are more prevalent among women. Inclusion and standardization of qualitative parameters along with quantitative measurements in echocardiography reports could help guide clinical decision-making, resolve discordant cases, and increase appropriate diagnostic confidence. Machine learning algorithms to categorize AS severity at the time of TTE and provider alert systems to enhance timely recognition of severe AS on TTE have shown an improvement in sex disparities in recognition of AS and appropriate referral to intervention.^30^^,^^31^ A validated artificial intelligence–automated alert system increased recognition of guideline-defined severe AS on echocardiography as compared with clinician interpretation, and more frequently recategorized women as compared with men as having severe AS primarily based on presence of smaller AVAs.^30^ The recent DETECT AS (Electronic Provider Notification to Facilitate the Recognition and Management of Severe Aortic Stenosis) randomized clinical trial showed equalization of AVR rates among men and women with severe AS with incorporation of electronic provider notifications (EPNs) following a diagnostic TTE for severe AS. Women in the EPN group were 20% more likely to undergo AVR as compared to usual care with achievement of equal rates of AVR among both sexes in the EPN group,^31^ including among low gradient severe AS phenotype.

### Study Limitations

Our study results should be interpreted in the context of some recognized limitations. ICD diagnosis codes were used as a surrogate marker for the time at which the clinical recognition of AS occurred following diagnostic TTE. This framework assumes that a diagnosis of AS was not acknowledged before or in the absence of an ICD code, and that it did not occur outside of our health care system. We recognize that an ICD code is a surrogate for clinical recognition and not confirmatory for the presence of disease; however, it acknowledges the findings which is essential for downstream confirmatory testing and/or candidacy for treatment. We also recognize that ICD codes do not capture AS disease severity or phenotype thereby limiting their clinical utility if used alone.^32^ Our study was not designed to evaluate contributors to low diagnostic rates including referral to cardiology, number of follow-up visits after TTE, and referral to AVR among this specific cohort. We used self-reported sex for our analysis which may not account for any gender-specific disparities that may exist in the context of AS care. We make associations between low rates of diagnosis in this study as a contributor to low rates of AVR; however, the symptomatic status of the patients in this cohort and therefore appropriateness of AVR indication cannot be inferred.

## Conclusions

We identified significant sex differences in the clinical recognition of and assignment of an ICD diagnosis code for AS among patients with moderate or greater AS on echocardiography. Increasing the clinical recognition and diagnosis of TTE-detected AS represents an easy to identify and modifiable point of care with the potential for impactful downstream effects on quality and equity of AS care. Future efforts are needed to dismantle barriers to care and to maximize the equitable diagnosis and treatment of AS for all.

Perspectives **COMPETENCY IN PATIENT CARE:** Women with moderate or greater AS by TTE are less likely as compared with men to receive a diagnosis of AS following diagnostic echocardiography.

**COMPETENCY IN SYSTEMS-BASED PRACTICE:** Low rates of recognition of significant AS warrant further investigation into standardized echocardiography reporting standards, artificial intelligence–based diagnostic algorithms, and automated EHR alert systems to aid in the accurate diagnosis and recognition of AS.

**TRANSLATIONAL OUTLOOK:** Further studies are needed to determine if an increase in awareness and recognition of AS diagnosis following TTE is associated with the increased use of appropriate treatment interventions and mitigation of disparities in treatment among women.

## Funding support and author disclosures

Dr Elmariah has received research grants from 10.13039/100000968American Heart Association (19TPA34910170), 10.13039/100000002National Institutes of Health (R01 HL151838), 10.13039/100006520Edwards Lifesciences, 10.13039/100016304Medtronic, 10.13039/100000046Abbott; and has received consulting fees from Edwards Lifesciences. Dr Crousillat has received consulting fees from Heartflow. All other authors have reported that they have no relationships relevant to the contents of this paper to disclose.

## References

[1] Badheka A.O., Singh V., Patel N.J. (2015). Trends of hospitalizations in the United States from 2000 to 2012 of patients >60 years with aortic valve disease. Am J Cardiol.

[2] Castaldi G., Matetić A., Bagur R. (2025). Sex-associated disparities in surgical and percutaneous management of aortic stenosis with severe features: retrospective analysis from the national readmission database. J Am Heart Assoc.

[3] Chaker Z., Badhwar V., Alqahtani F. (2017). Sex differences in the utilization and outcomes of surgical aortic valve replacement for severe aortic stenosis. J Am Heart Assoc.

[4] Williams M., Kodali S.K., Hahn R.T. (2014). Sex-related differences in outcomes after transcatheter or surgical aortic valve replacement in patients with severe aortic stenosis. J Am Coll Cardiol.

[5] Panoulas V.F., Francis D.P., Ruparelia N. (2018). Female-specific survival advantage from transcatheter aortic valve implantation over surgical aortic valve replacement: meta-analysis of the gender subgroups of randomised controlled trials including 3758 patients. Int J Cardiol.

[6] Deharo P., Cuisset T., Bisson A. (2021). Outcomes following aortic stenosis treatment (transcatheter vs surgical replacement) in women vs men (from a nationwide analysis). Am J Cardiol.

[7] Pellikka P.A., Kato N. (2024). Aortic stenosis risk and management. JACC Cardiovasc Imaging.

[8] Desmarais T., Dichiacchio L., Fanous H., Levy L., Pereira S.J., Jones T.L. (2024). Gender disparities in valvular heart disease and treatment in the aging population. Curr Geriatr Rep.

[9] Tribouilloy C., Bohbot Y., Rusinaru D. (2021). Excess mortality and undertreatment of women with severe aortic stenosis. J Am Heart Assoc.

[10] Voisine M., Hervault M., Shen M. (2020). Age, sex, and valve phenotype differences in fibro-calcific remodeling of calcified aortic valve. J Am Heart Assoc.

[11] Willner N., Prosperi-Porta G., Lau L. (2023). Aortic stenosis progression. JACC Cardiovasc Imaging.

[12] Cramariuc D., Rogge B.P., Lønnebakken M.T. (2015). Sex differences in cardiovascular outcome during progression of aortic valve stenosis. Heart.

[13] Nguyen V., Mathieu T., Melissopoulou M. (2016). Sex differences in the progression of aortic stenosis and prognostic implication. JACC Cardiovasc Imaging.

[14] Heidenreich P.A., Bozkurt B., Aguilar D. (2022). 2022 AHA/ACC/HFSA guideline for the management of heart failure: a report of the American college of cardiology/American heart Association Joint Committee on clinical practice guidelines. Circulation.

[15] Crousillat D.R., Amponsah D.K., Camacho A. (2022). Racial and ethnic differences in the clinical diagnosis of aortic stenosis. J Am Heart Assoc.

[16] Otto C.M., Nishimura R.A., Bonow R.O. (2021). 2020 ACC/AHA guideline for the management of patients with valvular heart disease. J Am Coll Cardiol.

[17] Gagne J.J., Glynn R.J., Avorn J., Levin R., Schneeweiss S. (2011). A combined comorbidity score predicted mortality in elderly patients better than existing scores. J Clin Epidemiol.

[18] Sjoberg D.D., Fei T. (2023). _tidycmprsk: competing risks estimation_. R package version 1.0.0. https://CRAN.R-project.org/package=tidycmprsk.

[19] Gerds T., Ohlendorff J., Ozenne B. (2023). _riskRegression: risk regression models and prediction scores for survival analysis with competing risks_. R package version 2023.12.21. https://CRAN.R-project.org/package=riskRegression.

[20] Pellikka P.A., Padang R., Scott C.G., Murphy S.M.E., Fabunmi R., Thaden J.J. (2022). Impact of managing provider type on severe aortic stenosis management and mortality. J Am Heart Assoc.

[21] Strange G., Stewart S., Celermajer D. (2019). Poor long-term survival in patients with moderate aortic stenosis. J Am Coll Cardiol.

[22] Brennan J.M., Lowenstern A., Sheridan P. (2021). Association between patient survival and clinician variability in treatment rates for aortic valve stenosis. J Am Heart Assoc.

[23] Li S.X., Patel N.K., Flannery L.D. (2022). Trends in utilization of aortic valve replacement for severe aortic stenosis. J Am Coll Cardiol.

[24] Lindman B.R., Fonarow G.C., Myers G. (2023). Target aortic stenosis: a national initiative to improve quality of care and outcomes for patients with aortic stenosis. Circ Cardiovasc Qual Outcomes.

[25] Ito S., Miranda W.R., Nkomo V.T., Lewis B.R., Oh J.K. (2022). Sex differences in LV remodeling and hemodynamics in aortic stenosis. JACC Cardiovasc Imaging.

[26] Clavel M.-A., Magne J., Pibarot P. (2016). Low-gradient aortic stenosis. Eur Heart J.

[27] Messika-Zeitoun D., Oh J.K., Topilsky Y., Burwash I.G., Michelena H.I., Enriquez-Sarano M. (2018). Low-gradient aortic stenosis: solving the conundrum using multi-modality imaging. Prog Cardiovasc Dis.

[28] Cramariuc D., Bahlmann E., Gerdts E. (2022). Grading of aortic stenosis: is it more complicated in women?. Eur Cardiol.

[29] Bienjonetti-Boudreau D., Fleury M.-A., Voisine M. (2021). Impact of sex on the management and outcome of aortic stenosis patients. Eur Heart J.

[30] Strange G.A., Feneley M.P., Prior D. (2024). Detection of severe aortic stenosis by clinicians versus artificial intelligence: a retrospective clinical cohort study. Am Heart J Plus Cardiol Res Pract.

[31] Tanguturi V.K., Abou-Karam R., Cheng F. (2025). Electronic provider notification to facilitate the recognition and management of severe aortic stenosis: a randomized clinical trial. Circulation.

[32] Strom J.B., Xu J., Sun T. (2022). Characterizing the accuracy of international classification of diseases, tenth revision administrative claims for aortic valve disease. Circ Cardiovasc Qual Outcomes.

